# Identify perceived strategies for promoting happiness among nurses: Protocol for a mixed-methods study

**DOI:** 10.1371/journal.pone.0354376

**Published:** 2026-07-23

**Authors:** Seyedeh Somayeh Kazemi, Mansour Ranjbar, Mohammad Rafighi

**Affiliations:** 1 Department of Health Education & Promotion, Faculty of Health, Mazandaran University of Medical Sciences, Sari, Iran; 2 Orthopedic Research Center, Sari Imam Khomeini Hospital, Mazandaran University of Medical Sciences, Sari, Iran; 3 Department of Education Development Center (EDC), Mazandaran University of Medical Sciences, Sari, Iran; 4 Department of Education and Human Resources Development, Mazandaran University of Medical Sciences, Sari, Iran; Khyber Medical University, PAKISTAN

## Abstract

**Background:**

Enjoyment at work and overall happiness are essential elements of a thriving workforce, particularly in high-pressure environments like healthcare. Nurses frequently experience occupational burnout and stress due to the demanding nature of their profession, which can adversely affect their performance, job satisfaction, and overall well-being. Understanding the factors that contribute to happiness in nursing is crucial, as fostering a positive work environment can lead to improved patient care, reduced turnover rates, and enhanced mental health among healthcare professionals.

**Objective:**

This study aims to systematically investigate the current state of happiness among nurses in order to identify perceived strategies for enhancing their well-being. By employing a mixed-methods approach, we will gather quantitative data on happiness levels and qualitative insights into the experiences and perceptions of nurses regarding their work environment. The ultimate goal is to develop evidence-based recommendations that can be implemented within healthcare settings to promote happiness and mitigate the effects of burnout and stress, thereby contributing to a healthier and more productive workforce.

**Methods:**

Utilizing a mixed-method protocol, the research consists of two distinct phases. The quantitative phase employs a two-stage sampling method to select participants from ten randomly chosen hospitals within a province, ensuring a representative sample. In the qualitative phase, snowball sampling will be utilized to achieve data saturation, allowing for a comprehensive understanding of the factors influencing happiness in nursing. Data collection will involve a range of instruments, including a demographic characteristics and occupational features checklist, the Oxford Happiness Questionnaire, and semi-structured interviews to gather in-depth insights. Data analysis will be conducted using SPSS-24 statistical software for quantitative data and MAXQDA for qualitative data, enabling a robust examination of the findings.

**Result:**

The study is expected to yield a comprehensive understanding of the levels and determinants of happiness among nurses. Quantitative data will provide statistical insights into the prevalence of happiness and its correlation with demographic and occupational factors. Qualitative findings are anticipated to uncover nuanced perspectives on the challenges and enablers of workplace happiness, offering valuable context to the quantitative results. Together, these findings will inform actionable recommendations for fostering a supportive work environment in healthcare settings.

**Conclusions:**

The anticipated outcomes of this study highlight the positive impact of workplace happiness on nurses’ health, job satisfaction, and overall quality of patient care. By identifying actionable perceived strategies to promote happiness in the workplace, this research aims to contribute to the development of supportive environments that enhance both staff well-being and patient outcomes. Ultimately, this study represents a vital step toward fostering a healthier, more engaged nursing workforce.

**Ethics approval:**

IR.MAZUMS.IMAMHOSPITAL.REC.1402.119

## 1. Introduction

### 1.1 Context and challenges

Happiness is often regarded as one of the most profound gifts of human existence. According to numerous experts, all human behaviors are ultimately aimed at achieving this elusive state [1]. Happiness not only serves as a fundamental measure of mental health but also brings a multitude of tangible benefits that extend beyond the individual. Research indicates that happiness can enhance physical health, reduce psychological distress, and even contribute to a longer lifespan. Empirical studies have consistently shown that happy individuals tend to experience a range of positive outcomes, including stronger supportive relationships, improved mental and physical health, and, consequently, increased longevity [2]. In the context of healthcare, the happiness of professionals—especially nurses—is paramount. Nurses are the backbone of the healthcare system, often serving as the primary point of contact for patients. Their well-being directly influences not only their job satisfaction but also the quality of care they provide. A happy nurse is more likely to exhibit compassion, empathy, and resilience, which are critical attributes in a demanding healthcare environment. Conversely, unhappiness among nurses can lead to burnout, decreased job performance, and ultimately impact patient outcomes. Despite the recognized importance of happiness in healthcare settings, many nurses face significant challenges that can hinder their well-being. Factors such as high workloads, emotional strain, and organizational pressures contribute to a stressful work environment. As a result, addressing the happiness of nurses is not merely a matter of individual well-being but a crucial component of fostering a sustainable and effective healthcare system. This underscores the need for targeted interventions aimed at enhancing happiness among nursing professionals, which can lead to improved job satisfaction and better patient care.

### 1.2. Background

Happiness is increasingly recognized as a vital component of health, as emphasized by the World Health Organization [3,4]. In many countries, happiness levels are measured as indicators of quality of life [5]. Ed Diener, a leading researcher in happiness studies, defines happiness as the experience of life satisfaction and enduring joy, with minimal unpleasant emotions such as grief or anger [6]“. It comprises several elements: an emotional component related to cheerful disposition, a social component linked to enjoyment of relationships and social support, and a cognitive component that influences how individuals process information, interpret experiences, and ultimately feel joy and optimism [7].

The World Happiness Report (2017–2019) highlights significant disparities in happiness across nations, with Finland ranking highest and Afghanistan lowest. The Islamic Republic of Iran ranks 118th among 153 countries, indicating a need for improvement, particularly compared to some Eastern Mediterranean countries like Saudi Arabia and Pakistan [8].

Despite the historical quest for happiness [9], research on this concept remains relatively new [10]. Happiness is crucial in all professions, especially nursing, where practitioners face unique challenges. Nurses are in constant contact with patients, requiring them to be compassionate and energetic—qualities closely tied to happiness [11]. However, they also encounter significant stressors, including exposure to patients’ pain, heavy workloads, and job dissatisfaction, which contribute to high levels of burnout [12]. A study conducted in New Zealand in 2020 reported that personal burnout prevalence was 60%, work-related burnout was 55%, and patient-related burnout was 19% [13]. The consequences of occupational stress for nurses include fatigue, lack of empathy, and job burnout [14,15]. These negative effects not only impact individual nurses but also extend to patients and healthcare organizations, leading to poor communication, dissatisfaction, and high staff turnover [15–17]. Despite the historical nature of these issues, the increasing prevalence of burnout suggests a need for innovative solutions.

Numerous studies have attempted to address burnout, yet evidence of successful and sustainable interventions remains limited [18]. The ongoing healthcare crisis has become normalized, necessitating radical transformative approaches to combat burnout [19]. One promising strategy is fostering happiness in the workplace. “Enjoyment in work” and happiness are essential for enabling healthcare professionals to positively impact their work goals and meaning [20,21], potentially serving as antidotes to burnout and stress [22].

As healthcare environments grow increasingly complex, the need for patient-centered, safe, and effective nursing care becomes more critical [21]. Developing healthcare settings that allow nurses to find meaning and enjoyment in their daily work is essential for workforce engagement and retention [21]. Despite the significance of happiness in professional contexts, research specifically addressing happiness among nurses remains limited [23], highlighting the necessity for further exploration into well-being within nursing literature. Addressing this gap, the present protocol responds to a pressing academic problem: while existing literature has extensively documented occupational burnout, stress, and turnover among nurses, there is a lack of positive, strengths-based approaches—specifically, the systematic identification of strategies to promote happiness rather than merely reducing negative states. Current evidence on sustainable interventions to enhance nurse well-being is scarce, with most studies adopting reactive rather than transformative frameworks. This study directly shifts the focus from burnout mitigation to happiness promotion, aligning with global health system priorities such as the Quadruple Aim—improving patient care, enhancing workforce health, reducing costs, and restoring meaning in work. By employing a rigorous mixed-methods design combining the Oxford Happiness Questionnaire and in-depth qualitative interviews, this research will provide statistical insights into the prevalence and correlates of happiness among nurses alongside rich, contextual understandings of their lived experiences. Ultimately, the findings will yield actionable, evidence-based recommendations for healthcare administrators and policymakers, thereby contributing to public health, health policies, and the existing body of knowledge in this vital area.

## 2. Materials and methods

This protocol describes a mixed-methods investigation approved by the Ethics Committee for Health Research Ethics of Mazandaran University of Medical Sciences (approval code: IR.MAZUMS.IMAMHOSPITAL.REC.1402.119). The study consists of two phases. The first phase involves a cross-sectional survey, and the second phase comprises semi-structured interviews. All procedures will be conducted in accordance with the ethical principles outlined in the Declaration of Helsinki and relevant national guidelines. Written informed consent will be obtained from all participants prior to their involvement in any study-related procedures, including both the quantitative survey and qualitative interviews. An overview of the different phases containing aims, methods, and participants are depicted in Table 1.

**Table 1:** The study overview.

Phases	Aim	Methods	Participants
**Phase 1**			
Quantitative study (Cross-sectional study)	Investigate happiness (Identifying happiness levels and key demographic and occupational variables that contribute to or detract from happiness in the workplace)	Checklist of demographic characteristics and occupational features Oxford Happiness Questionnaire	Nurses
**Phase 2**			
Qualitative study	- Exploring the complex factors influencing nurses’ happiness in their work environment - Identifying strategies to promote happiness	In-depth interviews	Nurses

This manuscript details the protocol for a study that has not yet initiated participant recruitment. The planned timeline for the study is as follows:

Recruitment for both the quantitative (cross-sectional) and qualitative (interview) phases is anticipated to begin in 20 May 2026 and is expected to be completed within 4 months (20 Sep 2026). Data collection is scheduled to run concurrently with recruitment and is projected to be finalized within 3 months (20 Des 2026). Data analysis and compilation of results are planned to be completed within 4 months after the end of data collection.

### 2.1 Study 1: Cross‑sectional survey

This study adopts a sequential explanatory mixed‑methods design. The rationale for this design is that the quantitative phase (cross‑sectional survey) will first identify the levels and correlates of happiness among nurses. Subsequently, the qualitative phase (semi‑structured interviews) will be used to explain, elaborate on, and provide contextual depth to the quantitative findings. This design is well‑suited to our aim of developing evidence‑based, actionable strategies, as the qualitative data will help interpret statistical patterns and uncover the mechanisms underlying the observed relationships. The primary aim of the first phase of this study is to conduct a cross-sectional investigation into the levels of happiness among nurses working in hospitals. This quantitative approach will involve the administration of standardized surveys to assess various dimensions of happiness, including emotional, social, and cognitive components. By collecting data from a diverse sample of nurses across different hospital settings, the study seeks to quantify happiness levels and identify factors that contribute to or hinder happiness in the workplace. The findings will provide a comprehensive overview of the current state of happiness among nurses, highlighting areas that require attention and intervention. To achieve this aim, the following research hypotheses will be tested:

1. There is a significant relationship between demographic variables (age, gender, marital status, family income) and nurses’ happiness scores.
2. There is a significant relationship between occupational variables (work experience, employment status, salary satisfaction, overtime hours, shift work) and happiness scores.
3. Nurses who participate in recreational and cultural activities (either at the workplace or outside of it) have higher happiness scores compared to nurses who do not participate in such activities.
4. Satisfaction with physical and mental health has a significant positive correlation with nurses’ happiness scores.
5. A regression model encompassing demographic and occupational variables will be able to significantly predict a portion of the variance in nurses’ happiness scores.

#### 2.1.1 Study setting.

The research will be conducted in hospitals affiliated with Mazandaran University of Medical Sciences. This setting is chosen to ensure a representative sample of nurses working in various clinical environments.

#### 2.1.2 Participants.

The target population for this study consists of nurses employed in hospitals affiliated with Mazandaran University of Medical Sciences.

#### 2.1.3 Inclusion criteria.

Participants must meet the following criteria:

a) Male and female nurses from each unit.
b) A minimum of 24 working hours per week.
c) At least three years of work experience in nursing.

#### 2.1.4 Exclusion criteria.

Participants will be excluded if they:

a) Express unwillingness to cooperate during the survey process.

#### 2.1.5 Data collection.

Data will be collected using the following instruments:

A. self-design checklist including demographic characteristics and occupational features.
B. The standard Oxford Happiness Questionnaire, which will be sent to participants online.

**A. Checklist of demographic characteristics and occupational features:** This checklist will include demographic characteristics and occupational features.

The demographic section will cover: Age, Gender, Number of children, Marital status, Educational background, Spouse’s employment status, Type of housing, Family income, Engagement in recreational or cultural activities, Satisfaction with physical health, Satisfaction with mental health.

The occupational features section will include: Official position of nurses, Employment status, Current department of the workplace, Level of interest in nursing at present, Satisfaction with salary, Number of overtime hours, Shift work, Engagement in recreational activities at the workplace.

**B. Oxford Happiness Questionnaire:** The Oxford Happiness Questionnaire consists of 29 items divided into six subscales: self-esteem, life satisfaction, efficiency, positive mood, sense of control, and mental health. Respondents will answer items on a six-point Likert scale ranging from “strongly disagree” to “strongly agree.” The scoring for the six scales ranges from 1 to 6, leading to a total score that ranges from 29 to 174. A higher score indicates greater happiness. The total score will be classified into three levels: less than 100 indicates low happiness, scores between 101–131 indicate medium happiness, and scores above 132 indicate high happiness [24,25]. The validity and reliability of the Persian version of the questionnaire have been confirmed in previous studies, with a reported Cronbach’s alpha of over 0.90 [24–26].

#### 2.1.6 Sample size.

The initial sample size was calculated using the formula for estimating a population mean: n=(Zα/2 × σ/d)2n=(Zα/2 × σ/d)2. Based on a previous similar study [27], the mean happiness score was 67.43 with a standard deviation (SD) of 17.28. Assuming a 95% confidence level (Zα/2 = 1.96Zα/2 = 1.96) and a margin of error (d) of 2 points, the calculated sample size was 287. However, to account for the two‑stage cluster sampling design where hospitals are first randomly selected, followed by the random selection of nurses within each hospital proportionate to the hospital’s nursing staff size a design effect (DE) will be incorporated into the sample size calculation. The design effect is computed using the formula DE = 1 + (m − 1) × ICC, where m represents the average cluster size (the number of participants per hospital) and ICC denotes the intra‑cluster correlation coefficient. Based on previous similar studies conducted in Iran examining health outcomes among healthcare workers, an ICC of 0.05 is assumed for happiness‑related variables among nurses within the same hospital. With an estimated average cluster size of 34 nurses per hospital (derived by dividing the total target sample by the number of hospitals), the design effect is calculated as 1 + (34 − 1) × 0.05 = 2.65.

#### 2.1.7 Randomization.

Sampling in the quantitative phase will be conducted in two stages. In the first stage, a list of all hospitals in the province (26 hospitals) will be prepared through Mazandaran University of Medical Sciences, from which 10 hospitals will be randomly selected. The names of the hospitals will be written on cards, placed in a box, mixed, and then 10 cards will be drawn one by one. In the second stage, participants will be selected from each hospital using random sampling based on the number of nurses employed in each facility. Each hospital will provide a list of nurses’ IDs to the study coordinator, who will code these identifiers and create a master list. The required number of samples will then be selected from this list using a random number table, after which the selected individuals will be contacted to assess their eligibility and willingness to participate.

#### 2.1.8 Statistical analysis.

In the first phase of the study, data will be analyzed using SPSS-24 and STATA/MP software. To account for the two-stage sampling design and potential clustering effects (nurses nested within hospitals), linear mixed-effects models will be employed with ‘hospital’ included as a random intercept. This approach will provide robust standard errors and correct estimates of variance. The intra-class correlation coefficient (ICC) will be calculated to assess the degree of clustering. For categorical variables, Rao-Scott chi-square tests will be used. A significance level of 0.05 will be maintained for all tests. The extent and pattern of missing data will be assessed using Little’s MCAR test. Data are assumed to be Missing at Random (MAR). Instead of mean imputation, Multiple Imputation by Chained Equations (MICE) will be used to handle missing values in both demographic and outcome variables, generating 20 imputed datasets. The results from these datasets will be pooled using Rubin’s rules. A sensitivity analysis will be conducted by comparing results from the complete-case analysis with those from the multiple imputation approach to ensure robustness.

The first phase of this study, a cross-sectional survey of happiness among nurses in hospitals affiliated with Mazandaran University of Medical Sciences, aims to provide a comprehensive understanding of the factors influencing nurses’ well-being. By employing standardized instruments, including the Oxford Happiness Questionnaire, this phase will quantify happiness levels and identify key demographic and occupational variables that contribute to or detract from happiness in the workplace. The findings are expected to highlight critical areas for intervention, which could lead to improved job satisfaction and overall well-being among nurses. Ultimately, this phase will lay the groundwork for subsequent qualitative research, facilitating a deeper exploration of the lived experiences of nurses and informing targeted strategies to enhance their happiness in the profession.

### 2.2 Study 2: Qualitative study

The second phase of this study will employ a qualitative approach to gain deeper insights into the experiences and perceptions of nurses regarding happiness in their work environment. Through in-depth interviews, this phase aims to explore the nuanced factors that influence happiness, such as workplace culture, support systems, and personal coping mechanisms. By capturing the voices and narratives of nurses, the qualitative study will identify specific strategies that can be implemented to enhance happiness and well-being in the nursing profession. This dual approach, combining quantitative and qualitative methods, will provide a holistic understanding of happiness in nursing and inform effective interventions to promote a more positive work environment. To guide this phase, the following primary and secondary qualitative research questions will be addressed:

The primary qualitative research question is: What are the lived experiences and perceptions of nurses regarding happiness in the workplace, and what factors, from their perspective, contribute to or hinder professional happiness?

Secondary Qualitative Research Questions include:

1. How do nurses define and describe happiness in the workplace?
2. What individual factors (e.g., mental health, lifestyle, coping skills) from the nurses’ perspective influence their happiness?
3. What organizational and workplace factors (e.g., organizational culture, managerial support, collegial relationships, human resources) are experienced as facilitators or barriers to professional happiness?
4. From the nurses’ perspective, what is the impact of happiness on professional performance, quality of patient care, and interactions with colleagues?
5. What practical strategies and solutions (at the individual, team, and managerial levels) are proposed to promote happiness in the nursing work environment?
6. What structural or cultural barriers within healthcare organizations impede the realization of professional happiness among nurses?

#### 2.2.1 Study setting.

The qualitative study will be conducted in various hospitals affiliated with Mazandaran University of Medical Sciences, providing a rich context for understanding the diverse experiences of nurses.

#### 2.2.2 Participants.

The participants will consist of professional nurses employed in the selected hospitals, ensuring a representative sample of perspectives from different nursing units. Participants for the qualitative phase will be purposively selected from those who completed the survey. Selection will be based on their happiness scores (e.g., nurses with very low, moderate, and very high scores) and key occupational variables (e.g., shift work, overtime hours, satisfaction with salary). This ensures that the qualitative sample is directly informed by, and linked to, the quantitative results.

#### 2.2.3 Inclusion criteria.

Participants must meet the following criteria:

a) Willingness to share their experiences regarding happiness in the workplace.
b) Ability to engage in the study with maximum diversity in terms of age, gender, and nursing specialties**.**

#### 2.2.4 Exclusion criteria.

Participants will be excluded if they:

a) Are unwilling to participate or share their experiences during the interviews.

#### 2.2.5 Data collection and interview procedures.

**A. Semi-structured interviews:** This qualitative exploratory-descriptive study aims to uncover the perspectives and experiences of professional nurses regarding happiness in the workplace. The study seeks to elucidate nurses’ experiences and viewpoints on happiness, its impact on their performance, and the identification of ways to enhance professional happiness in healthcare settings. To achieve this, semi-structured interviews will be conducted, with questions formulated based on the study’s objectives and a review of relevant literature. Preliminary analysis of the quantitative data will be used to refine the semi‑structured interview guide. For example, if the survey reveals that nurses with high overtime hours report significantly lower happiness, the interview questions will explore the specific experiences and contextual factors underlying this finding. The interviews will explore and understand the nurses’ experiences and perspectives on happiness and its implications for their professional performance. Insights, suggestions, ideas, and potential solutions will be gathered to contribute to enhancing professional happiness in healthcare environments. Data saturation will be ensured by continuing interviews until no new information emerges, thereby ensuring comprehensive coverage of a wide range of perspectives and experiences related to the study’s objectives. To maintain the validity and reliability of the data, interviews will be conducted in a tranquil environment, with note-taking and audio recording to ensure accuracy and thorough immersion in the data.

To ensure the validity and reliability of the data in the qualitative research, the study will incorporate measures based on the criteria established by Guba and Lincoln [28]. Four criteria will be utilized as standards of scientific rigor: credibility, transferability, dependability, and confirmability.

- **Credibility** will be established through member checking and prolonged engagement, fostering rapport and understanding of their experiences.
- **Transferability** will be achieved by employing maximum diversity in sampling techniques, allowing findings to be applicable across various contexts. Operationalization of maximum diversity includes variation based on age (age groups: 20–30, 31–40, 41–50+), gender (male and female), nursing specialty (e.g., emergency, ICU, medical-surgical), and shift type (fixed, rotating, night shift). Despite using snowball sampling as an auxiliary technique, the primary sampling strategy will be purposive sampling driven by the quantitative results to ensure this diversity from the outset.
- **Dependability** will be ensured by documenting similar situations for participants, promoting consistency in findings. An audit trail will be maintained, documenting all analytical decisions and methodological choices.
- **Confirmability** will be enhanced through reflexive memoing and external review by experts and integrating supplementary opinions into the data analysis process.

Furthermore, to ensure the reliability of the data, the study will utilize methods such as note-taking and recording personal reflections to enhance objectivity. The use of MAXQDA 10 software for text coding will demonstrate a systematic and rigorous approach to data management and analysis, reinforcing the reliability of the findings [28]. In conclusion, this study will utilize a rigorous methodology with semi-structured interviews to explore nurses’ professional happiness. The goal is to produce reliable findings that enhance the well-being and performance of nurses in healthcare settings.

### Interview implementation process

#### Interviewer identity, role, and training.

All semi-structured interviews will be conducted by the principal investigator (Seyedeh-Somayeh Kazemi). The interviewer has no supervisory or evaluative authority over the participating nurses, which will be clearly stated at the beginning of each interview to reduce potential power imbalance and social desirability bias. Prior to data collection, the interviewer will complete a 20-hour training workshop on qualitative interviewing techniques, including active listening, probing, reflexivity, and ethical conduct in sensitive workplace research.

#### Interview mode and approximate duration.

Interviews will be conducted face‑to‑face in a private, quiet room within the participants’ hospital such as a supervisor’s office or a meeting room to ensure confidentiality and minimize interruptions. Each interview is expected to last between 45 and 60 minutes, depending on participant engagement and the richness of the responses provided. No repeat interviews are planned; however, participants will be contacted for member checking if clarification or further elaboration on their responses is required.

#### Piloting of the interview guide.

The semi-structured interview guide will be piloted with five nurses who meet the inclusion criteria but will not be included in the main study sample. Piloting will assess question clarity, flow, cultural appropriateness, and estimated duration. Based on pilot feedback, ambiguous or redundant questions will be revised or removed. The final version of the interview guide will be submitted as supplementary material alongside the published protocol.

#### Transcription process.

All interviews will be audio-recorded using a digital voice recorder after obtaining explicit written consent. Recordings will be transferred to a password-protected computer and deleted from the recording device immediately after transfer. Verbatim transcription will be performed within 48 hours of each interview by a trained research assistant who is not involved in participant recruitment. Transcribers will sign a confidentiality agreement. Each transcript will be checked against the original audio recording by the principal investigator for accuracy. Identifying information (names, hospital names, specific units) will be removed or pseudonymized during transcription. Transcripts will not be returned to participants for correction, but participants will receive a summary of key themes for member checking as part of credibility enhancement.

#### 2.2.6 Sample size.

In the qualitative phase, sampling will be conducted using a snowball method, where selection will be voluntary and based on referrals from previous participants [29]. Although snowball sampling will be used as an auxiliary technique to reach additional information‑rich cases, the primary sampling strategy for the qualitative phase will be purposive sampling driven by the survey results. This ensures that the qualitative sample is not disconnected from Phase 1 but rather emerges directly from it. Next step, initial participants will be asked to recommend other potential interviewees, thereby expanding the sample size progressively. Written consent will be obtained from each participant prior to the interview.

#### 2.2.7 Statistical analysis.

Data analysis in the qualitative phase will follow a reflexive thematic analysis approach as outlined by Braun and Clarke [30]. This process begins with the researcher taking notes on initial ideas and reflections after each interview and concludes with the writing of the final report. The six phases of analysis include: (1) familiarization with the data, (2) generating initial codes, (3) generating initial themes, (4) reviewing and developing themes, (5) refining, defining, and naming themes, and (6) writing the report. Codes and themes will be generated from individual transcripts as well as from the entire dataset using MAXQDA software or manually. An inductive approach will be employed to develop themes, allowing for a thorough analysis of the data without imposing pre-existing frameworks or theories [30]. Themes will be reviewed and discussed with the second author to ensure a comprehensive grouping of common codes, patterns, and concepts, thereby enhancing the robustness of the analysis. To ensure a complete audit trail, all coding decisions, theme development stages, and reflexive memos will be documented and stored within MAXQDA or manually. Additionally, the date and time of analysis sessions, different versions of the code tree, and the researcher’s personal reflections on how each theme was derived will be recorded.

The second phase of this study employs qualitative methods to explore the complex factors influencing nurses’ happiness in their work environment. By conducting in-depth interviews, the research will reveal insights that can guide effective interventions and enhance overall job satisfaction and well-being among nurses. After both phases are completed, the quantitative and qualitative results will be compared, contrasted, and synthesized. A joint display (matrix or side‑by‑side table) will be created to show how qualitative themes confirm, expand upon, or contradict the statistical findings. This integrated interpretation will directly inform the final set of recommended strategies for promoting happiness among nurses.

## 3. Ethics

The Research Ethics Committees of Imam- Mazandaran University of Medical Sciences Approved the study (IR.MAZUMS.IMAMHOSPITAL.REC.1402.119). Written informed consent will be obtained from all participants prior to their involvement in any study-related procedures, including both the quantitative survey and qualitative interviews. Participants will be fully informed about the purpose of the study, the voluntary nature of their participation, and their right to withdraw at any time without any negative consequences. Withdrawal will not affect their employment, education, or access to services. Confidentiality will be strictly maintained throughout the study; all personal identifiers will be removed or coded, and data will be stored securely. Data protection procedures include password-protected electronic files, limited access to authorized researchers only, and secure physical storage of any paper-based records. All data will be anonymized prior to analysis and reported only in aggregate form to prevent individual identification.

## 4. Result

The anticipated results of this study include a detailed understanding of the current state of happiness among nurses and the identification of factors that significantly influence their well-being. Quantitative data, collected through validated instruments such as the Oxford Happiness Questionnaire, are expected to reveal patterns and correlations between happiness levels and various demographic and occupational characteristics, including age, years of experience, job roles, and shift patterns. These findings aim to provide a statistical foundation for understanding the prevalence and distribution of happiness in the nursing profession.

In the qualitative phase, semi-structured interviews are anticipated to yield rich, contextual data, uncovering deeper insights into the lived experiences of nurses. Thematic analysis is expected to highlight critical themes, such as the role of workplace culture, management support, peer relationships, opportunities for professional growth, and the impact of work-life balance on happiness.

By synthesizing the quantitative and qualitative findings, the study aims to construct a holistic framework for understanding happiness in nursing. This comprehensive approach is expected to inform actionable strategies tailored to address both systemic and individual factors that impact happiness. These strategies will contribute to fostering a positive work environment, ultimately enhancing nurse retention, reducing burnout, and improving patient care outcomes.

## 5. Discussion

A key feature of this study is its focus on promoting happiness in the workplace, particularly among nurses. By investigating the factors that contribute to happiness and identifying perceived strategies for enhancement, this research aims to foster a more positive work environment. The primary framework will be a mixed-methods approach, integrating quantitative data with qualitative insights gathered through in-depth interviews. This experience-based methodology will allow for a comprehensive understanding of the unique challenges and opportunities that nurses face in their professional lives. By capturing the nuanced experiences and perceptions of nurses, the study seeks to highlight the importance of workplace culture, support systems, and personal coping mechanisms in shaping their overall happiness. The findings are expected to inform targeted interventions that not only enhance job satisfaction but also improve patient care outcomes by fostering a more engaged and motivated nursing workforce. Ultimately, this research aspires to contribute to the broader discourse on well-being in healthcare settings, emphasizing the critical role of happiness in promoting both individual and organizational success.

### 5.1 Strengths and limitations

#### Strengths of this study include.

1. Significance of the Topic: The research is important as it addresses the well-being of nurses, a crucial aspect of healthcare settings.
2. Mixed-Methods Approach: Using a mixed-methods design allows for a comprehensive understanding of happiness levels and perceived strategies for improvement.
3. Potential for Practical Implications: The findings could lead to the development of interventions and policies to enhance nurses’ well-being and job satisfaction.

#### Limitations include.

1. Sampling Bias: There might be limitations in the generalizability of the results if the sample is not representative of the broader population of nurses.
2. Self-Report Measures: Reliance on self-report measures for assessing happiness may introduce response bias and lack of objectivity.
3. Time and Resource Constraints: Conducting a mixed-methods study can be resource-intensive and time-consuming, which may limit the scope and depth of the research.

Overall, we will try to address these weaknesses through careful sampling strategies, methodological rigor, and adequate resource allocation can enhance the quality and validity of the research on investigating happiness in nurses.

## Supporting information

S1 File STROBE checklist. (DOC)
